# Evolution, functional differentiation, and co-expression of the *RLK* gene family revealed in Jilin ginseng, *Panax ginseng* C.A. Meyer

**DOI:** 10.1007/s00438-018-1425-6

**Published:** 2018-02-21

**Authors:** Yanping Lin, Kangyu Wang, Xiangyu Li, Chunyu Sun, Rui Yin, Yanfang Wang, Yi Wang, Meiping Zhang

**Affiliations:** 1 College of Life Science, Jilin Agricultural University, 2888 Xincheng Street, Changchun, 130118 Jilin China; 2 Research Center of Ginseng Genetic Resources Development and Utilization, 2888 Xincheng Street, Changchun, 130118 Jilin China; 3 College of Chinese Medicinal Materials, Jilin Agricultural University, Changchun, 130118 Jilin China

**Keywords:** Gene family, Receptor-like protein kinase (RLK), *Panax ginseng*, Gene family functional differentiation, Gene family expression correlation

## Abstract

Most genes in a genome exist in the form of a gene family; therefore, it is necessary to have knowledge of how a gene family functions to comprehensively understand organismal biology. The receptor-like kinase (RLK)-encoding gene family is one of the most important gene families in plants. It plays important roles in biotic and abiotic stress tolerances, and growth and development. However, little is known about the functional differentiation and relationships among the gene members within a gene family in plants. This study has isolated 563 *RLK* genes (designated as *PgRLK* genes) expressed in Jilin ginseng (*Panax ginseng* C.A. Meyer), investigated their evolution, and deciphered their functional diversification and relationships. The *PgRLK* gene family is highly diverged and formed into eight types. The LRR type is the earliest and most prevalent, while only the Lec type originated after *P. ginseng* evolved. Furthermore, although the members of the *PgRLK* gene family all encode receptor-like protein kinases and share conservative domains, they are functionally very diverse, participating in numerous biological processes. The expressions of different members of the *PgRLK* gene family are extremely variable within a tissue, at a developmental stage and in the same cultivar, but most of the genes tend to express correlatively, forming a co-expression network. These results not only provide a deeper and comprehensive understanding of the evolution, functional differentiation and correlation of a gene family in plants, but also an *RLK* genic resource useful for enhanced ginseng genetic improvement.

**Electronic supplementary material:**

The online version of this article (10.1007/s00438-018-1425-6) contains supplementary material, which is available to authorized users.

## Introduction

The majority of protein-encoding genes exist in multiple copies or the form of a gene family in the plant genomes (Schnable et al. 2009; Zhang et al. 2010; Karlgren et al. 2011). For instance, rice and *Arabidopsis* have 41,908 and 31,135 genes that contain 13,055 and 10,193 gene families (Schnable et al. 2009), respectively. Zhang et al. (2010) showed that the numbers of the gene members of a gene family vary by multiple fold not only between intrageneric species, but also within a species. Importantly, Zhang et al. (2010) found that the variation of the gene family size is significantly associated with the genetic variation of biological traits and plays important roles in plant variation, evolution and biology. Therefore, it is significant to understand the biology of gene families to comprehensively understand plant biology as a whole. Although several gene families, such as the nucleotide binding site (NBS)-encoding gene family (Monosi et al. 2004; Zhou et al. 2004; Tan et al. 2007; Kohler et al. 2008; Li et al. 2010; Lozano et al. 2012; Wan et al. 2012, 2013) and the receptor-like protein kinase (RLK)-encoding gene family (Zhang et al. 2010; Shiu et al. 2004), have been extensively studied, these studies were focused on structural and phylogenetic analyses of the gene families. Little is known about how the gene members of a gene family are related in functionality and in activity.

The *RLK* gene family represents a large gene family. In the dicot plant model species, *Arabidopsis thaliana*, at least 610 *RLK* genes were identified, accounting for nearly 2.5% of its protein-encoding genes (Shiu et al. 2004). In the monocot plant model species, rice, the number of *RLK* genes is doubled, consisting of more than 1,130 *RLK* genes (Shiu et al. 2004; Zhang et al. 2010). The first plant *RLK* gene was identified in maize (Walker and Zhang 1990). Stone and Walker (1995) showed that most *RLK* gene-encoding proteins consist of a ligand-identifying domain, a signal-accepting extracellular domain, a transmembrane domain and an intracellular domain with kinase activity. *RLK* genes have been shown to be involved in a wide range of biological processes, including growth and development, disease resistance, hormone perception and self-incompatibility (Morris and Walker 2003; Shiu et al. 2004; Afzal et al. 2008; Walia et al. 2009; Liebrand et al. 2013; Zhang et al. 2015). Among all the RLKs, the largest class of extracellular motifs is the leucine-rich repeats (LRRs), which often participate in protein–protein interactions (Kobe and Deisenhofer 1994; Bouwmeester et al. 2011; Gou et al. 2010; Sakamoto et al. 2012). The second largest class is sugar-binding motifs or lectins (Weis and Drickamer 1996). Epidermal growth factor (EGF)-like repeats, tumor necrosis factor receptor (TNFR)-like and other motifs were also found in plant RLKs (Becraft et al. 1996).

Ginseng, *Panax ginseng* C.A. Meyer, has been traditionally known in the Orient, particularly in China, Korea and Japan, as a medicinal herb. Chinese ginseng is mainly grown in Jilin Province, China, and known as Jilin ginseng. Medicinal research showed that ginseng has several bio-activities, including improving brain function, relieving pain, anti-tumor activity, and enhancing the immune system (Popovich et al. 2012). However, ginseng production is subjected to serious biotic and abiotic stresses. Although the *RLK* gene family is shown to play significant roles in combating these stresses (Morris and Walker 2003; Afzal et al. 2008; Walia et al. 2009; Liebrand et al. 2013), no *RLK* genes have been identified and reported in ginseng. This study not only isolated *RLK* genes expressed in Jilin ginseng and investigated the evolution of the *PgRLK* gene family, importantly, but also used this gene family as a model system to decipher the functional diversification and activity relationship among the gene members of the gene family. Therefore, these results provide not only an *RLK* genic resource necessary for ginseng genetic improvement and breeding, but also genome-wide new insights into how the genes within a gene family are related in functionality and activity in plants.

## Materials and methods

### Database resources

A Jilin ginseng transcriptome database consisting of 248,993 transcripts was used for this study. This comprehensive database of *P. ginseng* was developed from 14 tissues of a 4-year-old Jilin ginseng plant, including fiber root, leg root, main root epiderm, main root cortex, rhizome, arm root, stem, leaf peduncle, leaflet pedicel, leaf blade, fruit peduncle, fruit pedicel, fruit flesh, and seed (Wang et al. 2015). Using such a database would maximize the identification of the *RLK* genes expressed in ginseng. Moreover, the transcriptome databases previously developed from the roots of 5-, 12-, 18- and 25-year-old plants of Jilin ginseng (Wang et al. 2015) were also used. In addition, included were the database resources of the transcriptomes derived from the roots of 4-year-old plants of 42 Jilin ginseng farmers’ cultivars (coded from S1 to S42) collected from Jilin Province, China, representing the major origin and diversity center of *P. ginseng* (Yin et al. 2017). The detailed quantification of the expressions of the *PgRLK* genes and their transcripts were previously described (Wang et al. 2015; Yin et al. 2017).

### Identification of *PgRLK* genes and transcripts

Since no *RLK* genes isolated from *P. ginseng* were found in GenBank, we first identified the *RLK* genes from 19 other plant species in GenBank (http://www.ncbi.nlm.nih.gov/) and used as the starting materials for ginseng *RLK* gene identification. These 19 species included *Arabidopsis thaliana, Glycine max, Oryza sativa, Zea mays, Solanum lycopersicum, Solanum peruvianum, Solanum tuberosum, Daucus carota, Setaria italic, Cicer arietinum, Medicago truncatula, Cucumis sativus, Vitis vinifera, Populus trichocarpa, Populus nigra, Theobroma cacao, Cardamine flexuosa, Prunus mume* and *Citrus sinensis*. The *RLK* gene nucleotide sequences of these 19 plant species were translated in silico into putative protein sequences and comparatively analyzed. Their protein conservative domains were searched using the Vector NTI Advance software. These conservative sequences were then used as queries to search the *P. ginseng* transcriptome database of 248,993 transcripts with *E* value ≤ l.0e-04 using tblastn online at NCBI (http://blast.ncbi.nlm.nih.gov/Blast.cgi). The transcripts of ginseng containing the sequences having 50% or higher similarity to the RLK conservative domains were selected as the ginseng *RLK* transcript candidates. Therefore, the maximum number of genes potentially containing the RLK domains was included in the putative *RLK* gene list. These ginseng *RLK* transcript candidates were further subjected to annotation using Blast2GO (Ashburner et al. 2000) to determine which of them are actual *RLK* genes. The ginseng *RLK* transcript candidates that were annotated into *RLK g*enes were defined as *P. ginseng RLK* (*PgRLK*) gene transcripts. The genes from which the transcripts were derived were directly identified from the *P. ginseng* transcriptome database based on their gene IDs determined by the Trinity software (Grabherr et al. 2011; Haas et al. 2013).

### GO (gene ontology) functional categorization and enrichment analysis of the *PgRLK* transcripts

Because each gene may be alternatively spliced into multiple transcripts that are likely translated into different proteins having different biological functions, we categorized in silico the functions of the *PgRLK* genes based on their transcripts. The Blast2GO software (Ashburner et al. 2000) was used to categorize the functions of the *PgRLK* transcripts. The GO functional categorization of the transcripts of the entire transcriptome database was used as the background control or theoretical number of transcripts categorized into each subcategory (Level 2) for enrichment analysis of the *PgRLK* transcripts. The enrichment of the number of *PgRLK* transcripts categorized into each subcategory was determined by Chi-square test.

### Classification of the *PgRLK* genes

The *PgRLK* genes were subjected to NCBI-CD search and classified as follows. If an *RLK* gene was characterized with a leucine-rich repeat (LRR) motif and annotated into LRR receptor-like protein kinase, it was classified into “LRR type”. If an *RLK* gene was characterized with an S-locus, PAN or B-lec motif and annotated into s-domain class, it was classified into “S type”. If an *RLK* gene was characterized with a LysM motif and annotated into lysin motif receptor-like kinase, it was classified into “LysM type”. If an *RLK* gene was characterized with a lectin motif, it was classified into “Lec type”. If an *RLK* gene was annotated into calcium calmodulin-regulated receptor-like kinase, it was classified into “cal type”. If an *RLK* gene was annotated into cysteine-rich receptor-like protein kinase, it was classified into “cys type”. If an *RLK* gene was annotated into perk1-like protein kinase, it was classified into “PERK type”. If an *RLK* gene was annotated into protein kinase family protein, it was classified into “pro type”.

### Motif prediction of the *PgRLK* transcripts

The open reading frames (ORFs) of the *PgRLK* transcripts were searched using the ORF Finder at NCBI (http://www.ncbi.nlm.nih.gov/gorf/gorf.html). The *PgRLK* transcripts that had complete ORFs were further subjected to protein domain prediction using Pfam (http://pfam.sanger.ac.uk/) (Finn et al. 2014) and analyzed with the online software, MEME (multiple EM for motif elicitation) (http://meme.sdsc.edu/meme/cgi-bin/meme.cgi) for their motifs (Bailey et al. 2006). For this analysis, the maximum number of motifs was set to 5, motif length was set to 20–35 amino acids, and other parameters were used as default.

### Phylogenetic analysis of the *PgRLK* gene family

The transcripts that had the longest sequences and complete ORFs, one from each *PgRLK* gene ID, were selected and used for phylogenetic analysis of the *PgRLK* gene family. Therefore, a total of 136 *PgRLK* genes were selected for the phylogenetic analysis of the *PgRLK* gene family. In addition, 31 *RLK* genes from other plant species, with six or seven *RLK* genes randomly selected from each of the species, *S. lycopersicum, S. tuberosum, D. carota, O. sativa* and *A. thaliana*, were used as the evolutionary control of the *PgRLK* gene family. The putative protein sequences of the *PgRLK* genes were used for the analysis. The transcript sequences were translated into amino acid sequences, aligned with each other and constructed into a phylogenetic tree using MEGA6 (Tamura et al. 2013) with the maximum likelihood method. The phylogenetic tree of the *PgRLK* genes was also constructed using the ClustalX2 2.1 software (Larkin et al. 2007) with the neighbor-joining method (Saitou and Nei 1987). The trees were constructed with 10,000 bootstrap replications. The online software Evolview was used for landscaping and visualization of the gene family tree (Zhang et al. 2012).

### Expression and network analysis of the *PgRLK* transcripts

The R programming language and software (http://www.r-project.org/) were used to calculate Spearman’s correlation coefficients. The heatmaps were constructed using the R programming language and software, and the gene co-expression networks were constructed using the BioLayout Express^3D^ Version 3.2 software (Theocharidis et al. 2009).

## Results

### Identification of *PgRLK* genes

To identify the *PgRLK* genes in ginseng, we first conducted a search for *PgRLK* genes in GenBank (http://www.ncbi.nlm.nih.gov/), but no *PgRLK* gene was found. Therefore, we downloaded the *RLK* gene nucleotide sequences from 19 other plant species and used them as queries to conduct BLAST search against each other. Seven conserved domains were identified in the putative proteins of these *RLK* genes, including EKSDVYSFGVVLLE, LQHRNLVKLLGFC, IIHRDVKSNNILLD, GGFGPVYKGVL, IAGTYGYIAPEYA, LVYEYMENRSLASILF, and/or AKIADFGLAKLL. Then, we searched a transcriptome database of Jilin ginseng consisting of 248,993 transcripts developed from 14 tissues of a 4-year-old ginseng plant (Wang et al. 2015) using these conserved domains. Two thousand seven hundred and nineteen sequences were identified from the ginseng transcriptome database at a similarity of 50% or higher. The relaxed stringency allowed the identification of a maximum number of putative *RLK* transcripts from the database. To finally confirm whether these sequences were actual *RLK* genes, we further annotated them. Of the 2,719 putative *RLK* transcripts, 1364 were described as RLK and 4 were described as “NA” in the NCBI nr protein database. After eliminating the redundant sequences and the four “NA” transcripts, 964 *RLK* transcripts were obtained and designated as *PgRLK* transcripts (Table S1). These 964 *PgRLK* transcripts were derived from 563 *PgRLK* genes, as defined by the Trinity software (Grabherr et al. 2011; Haas et al. 2013) according to Wang et al. (2015). Each *PgRLK* gene was nominated by suffixing *PgRLK* with three digits and the different transcripts alternatively spliced from the same gene were named by suffixing the gene name with “-two additional digits” (Table S1).

To further verify the assembly of the *PgRLK* transcripts, we conducted the alignment of the 964 *PgRLK* transcripts to the ginseng genome sequence draft of Xu et al. (2017) using parameters of query cover ≥ 90%, identity ≥ 90% and *P* ≤ 1.0E-06. Consequently, 767 (79.56%) of the 964 *PgRLK* transcripts were aligned to the ginseng genome sequence draft. Since the failure of aligning the remaining 197 *PgRLK* transcripts to the ginseng genome sequence draft could be attributed to multiple reasons, including the transcript improper assembly, the ginseng genome draft assembly errors and/or gaps, and/or genetic diversity between the DNA source for the genome draft (Xu et al. 2017) and the RNA source for the transcript assembly (Wang et al. 2015), we conducted further alignment analysis by lowering the query cover of the alignment from 90 to 20% (the least length = 40 bp because the shortest transcript was 200 bp long), which is shorter than the 100-nucleotide reads for the RNA sequencing, without changes of the identity and *P* value. Consequently, none of the unaligned 197 *PgRLK* transcripts could be properly aligned to the genome draft, suggesting that the alignment failure of the 197 *PgRLK* transcripts to the ginseng genome draft largely resulted from the gaps of the genome draft. Furthermore, because transcripts are much more readily assembled accurately than genomes due to their much less complexity than genomes, the *PgRLK* transcripts were properly assembled.

### Motif identification and phylogeny of the *PgRLK* gene family

**Fig. 1:**
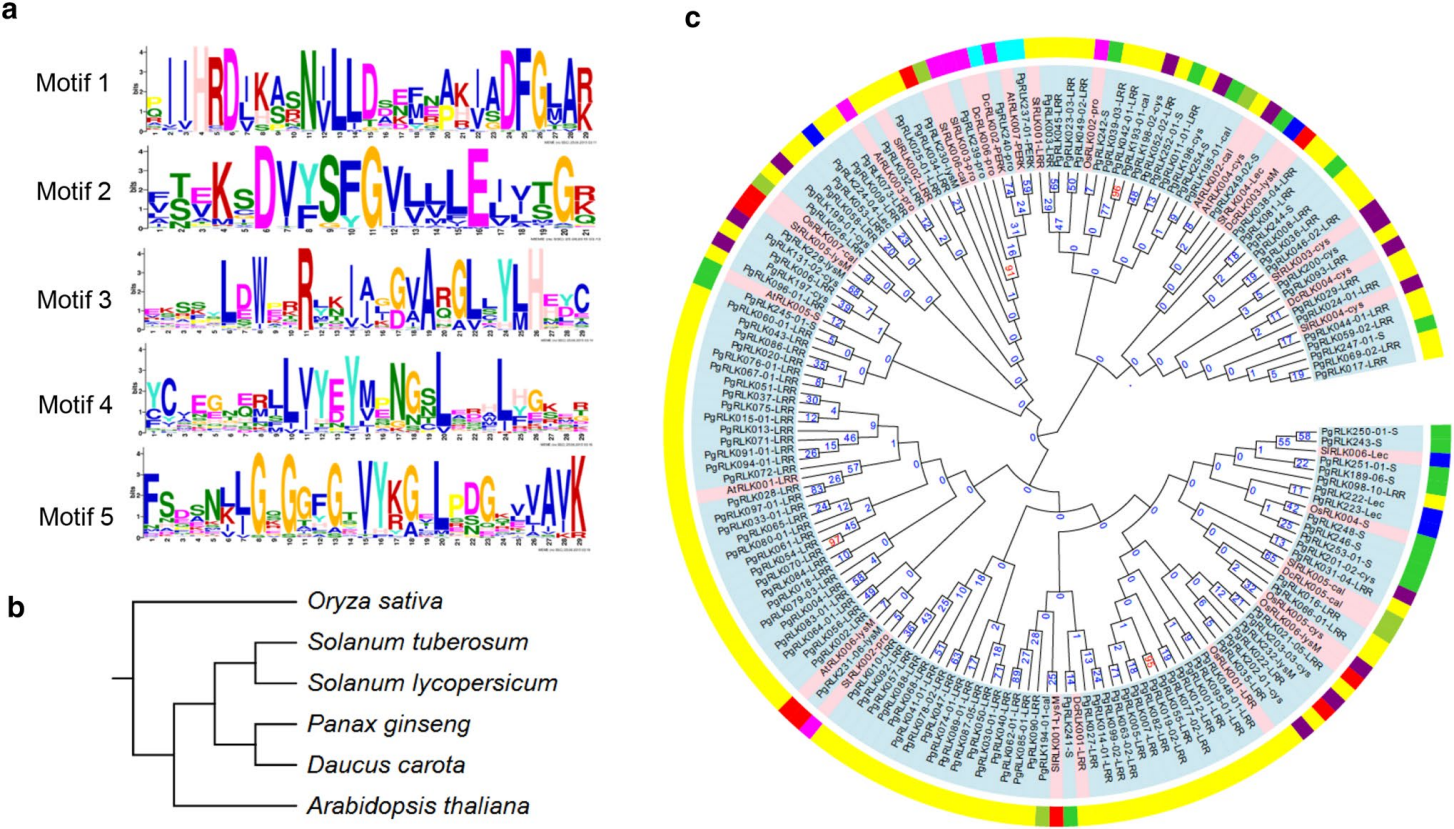
Conservative and phylogenetic analyses of the *PgRLK* genes. **a** Conservative domains of the putative proteins of *PgRLK* genes. **b** The phylogenetic position of *P. ginseng* in the seed plant phylogenetic tree. **c** Phylogenetic analysis of the *PgRLK* genes with the *RLK* genes selected from other plant species. The phylogenetic tree was constructed using MEGA6 (Tamura et al. 2013) with the maximum likelihood method. The number for each branch represents its bootstrap confidence presented by percentage (%) from 10,000 bootstrap replications. *At, Arabidopsis thaliana; Os, Oryza sativa; Sl, Solanum lycopersicum; Dc, Daucus carota; St, Solanum tuberosum*. The eight types of the *PgRLK* family are indicated by different colors: LRR type, yellow; cal type, light green; S type, lime green; LysM type, red; cys type, purple; PERK type, cyan; pro type, fuchsia; and Lec type, blue

We searched the 964 *PgRLK* transcripts for their ORFs using the ORF Finder at NCBI. Of these 964 transcripts, 724 were found to have complete ORFs. Therefore, the 724 transcripts were analyzed with Pfam (http://pfam.sanger.ac.uk/) to search for conserved domains. The proteins of 588 of the *PgRLK* transcripts were found to have one or more of the seven complete conserved domains identified in other plant species; therefore, they were further analyzed by MEME (multiple EM for motif elicitation) (http://meme.sdsc.edu/meme/cgi-bin/meme.cgi). Since the maximum number of motifs was set to five for this analysis, five conserved motifs were identified (Fig. 1a) for the vast majority of the *PgRLK* transcripts (Table S2). Moreover, we classified these 588 transcripts into eight types, *PgRLK-*LRR, *PgRLK-*cal, *PgRLK-*cys, *PgRLK*-Lec, *PgRLK*-LysM, *PgRLK*-PERK, *PgRLK*-pro and *PgRLK*-S (Table 1 and Table S3).

**Table 1:** Classification of the *PgRLK* genes based on their putative protein domains

Type	Letter code	No. of genes	No. of transcripts
Leucine-rich repeat	*PgRLK-*LRR	192	388
Calcium calmodulin regulated	*PgRLK-*cal	3	15
Cysteine rich	*PgRLK-*cys	26	89
Lectin-like	*PgRLK*-Lec	7	10
Lysin motif	*PgRLK*-lysM	8	25
Perk1-like	*PgRLK*-PERK	2	7
Protein kinase	*PgRLK*-pro	2	2
s-domain class	*PgRLK*-S	35	52
Total		275	588

RNA alternative splicing analysis of the Jilin ginseng transcriptome assembly showed that these 588 *PgRLK* transcripts were derived from 275 *PgRLK* genes (Table 1). Therefore, we randomly selected 136 *PgRLK* genes representing all eight types of the *PgRLK* gene family from the 275 *PgRLK* genes (Table 1) using the longest transcript as a representative for each gene and constructed the phylogenetic tree of the gene family. Thirty-one *RLK* genes, with six or seven *RLK* genes per species, identified from *Arabidopsis thaliana, Oryza sativa* and three ginseng-related species in GenBank (Fig. 1b and Table S4), were used as evolutionary controls. The phylogenetic tree of the *PgRLK* gene family and the *RLK* genes from the outgroup species were constructed with the maximum likelihood method and the neighbor-joining method, respectively, with 10,000 bootstrap replications. Figure 1c shows the phylogenetic tree of the *PgRLK* genes constructed using the maximum likelihood method and Fig. S1 shows the phylogenetic tree of the *PgRLK* genes using the neighbor-joining method. *Oryza sativa* is clustered into the basal node of the species phylogenetic tree (Fig. 1b), which is quite distantly related to the dicot plant, *P. ginseng*; however, the seven *OsRLK* genes from this monocot species, along with those from the dicot species (*A. thaliana*), were clustered into different clusters of the *PgRLK* gene family phylogenetic trees (Fig. 1c, Fig. S1). This result indicated that the *PgRLK* gene family originated before separation of dicot plants from monocot plants. Some of the *PgRLK* gene members belonging to the same type of the gene family tended to be clustered in the *PgRLK* gene family trees (Fig. 1c, Fig. S1). When the gene family trees (Fig. 1c, Fig. S1) were compared to the species tree (Fig. 1b), the LRR type of the *PgRLK* gene family (Table 1) seemed to be the most diverged and has the earliest origin, followed by the S and cys types, and then the LysM and cal types. The PERK, Lec and pro types originated most recently, but only the Lec type originated after *P. ginseng* evolved (Fig. 1c, Fig. S1).

### Functional differentiation of the *PgRLK* gene family

**Fig. 2:**
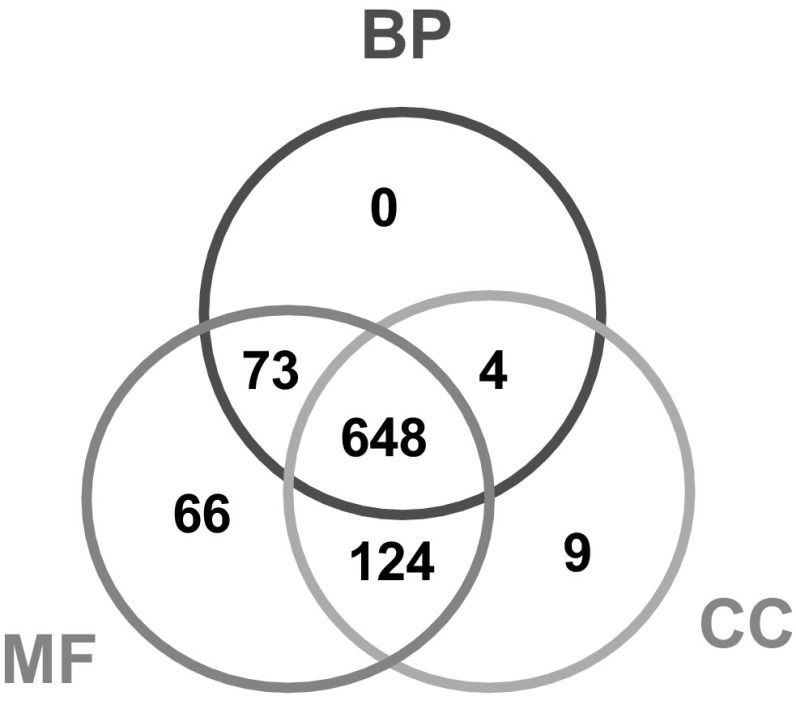
Venn diagram of the functional categorization of the *PgRLK* transcripts. *BP* biological process, *MF* molecular function, and *CC* cellular component

**Fig. 3:**
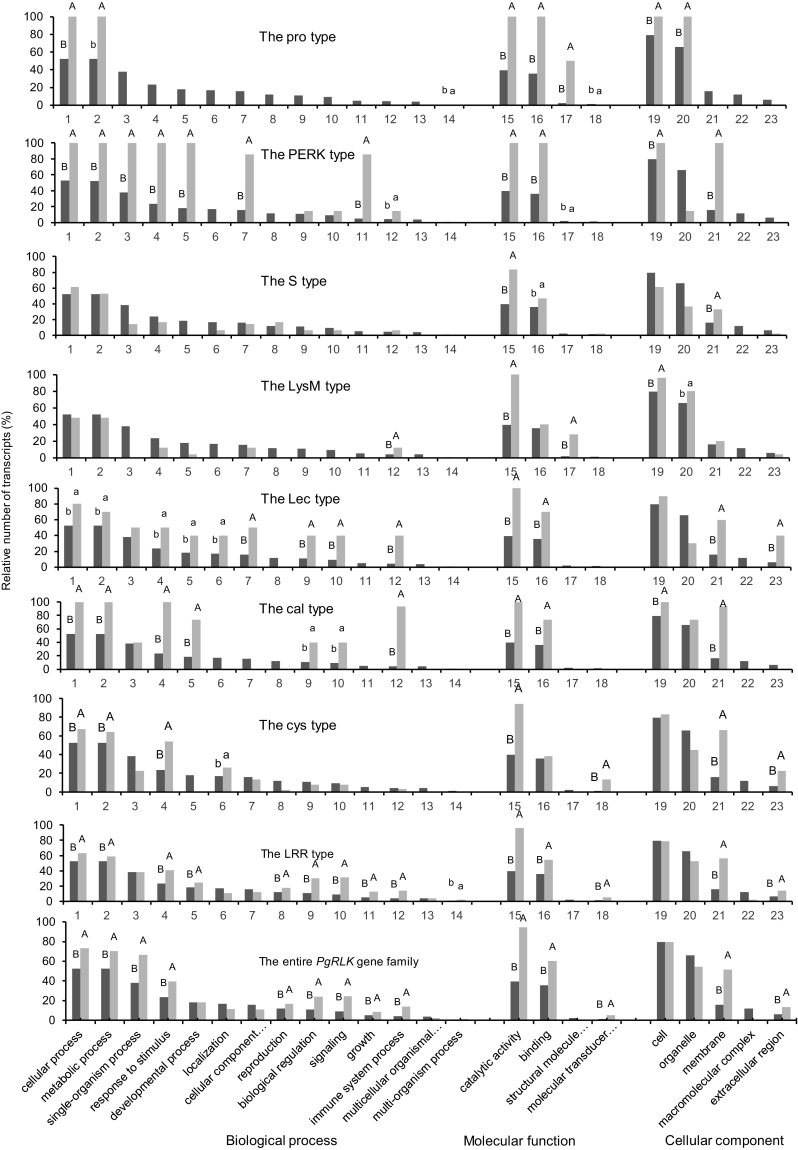
The GO functional categorization and enrichment of the *PgRLK* gene family and its eight types. The GO functional categorization of all genes expressed in the 14 tissues of a 4-year-old plant was used as the background control. Red bars indicate the background control and light blue bars indicate the GO functional categorization of the *PgRLK* gene family and its eight types. Capital letters, significant at *P* ≤ 0.01; small letters, significant at *P* ≤ 0.05; no letters, not significant

Since the genes of a gene family are congenetic and share similar sequences or conserved domains, it is often expected that the genes from a gene family have similar functions. To test this hypothesis and gain an insight into the functional relationships among the genes from a gene family, we categorized in silico the functions of the *PgRLK* genes using the Gene Ontology (GO) terms (Ashburner et al. 2000). The 964 *PgRLK* transcripts were used for the analysis because different transcripts alternatively spliced from the RNA of a single gene may have different biological functions. The results showed that 924 of these 964 transcripts were categorized into all three primary GO functional categories, biological processes (BP), molecular function (MF) and cellular components (CC) (Fig. 2 and Table S5). Of the 924 *PgRLK* transcripts, 648 (70%) had functions in all three primary categories, 201 (22%) had functions in two of the three primary categories and only 75 (8%) had functions in a single primary functional category. Furthermore, the 924 transcripts were categorized into 23 subcategories at Level 2, covering 64% of all 36 subcategories into which all ginseng gene transcripts expressed in 14 tissues were categorized (Wang et al. 2015). These included 14 BP subcategories, 4 MF subcategories and 5 CC subcategories (Fig. 3). Enrichment analysis showed that 14 of the 23 subcategories were significantly enriched relative to those of genome-wide gene transcripts (*P* ≤ 0.05). This result indicated that the functions of the genes of the *PgRLK* gene family have substantially differentiated as they evolved. Moreover, the functions of genes of each type of the *PgRLK* gene family have also greatly differentiated, even though they were determined to be within a particular cluster in the phylogenetic tree of the gene family (Fig. 3). Nevertheless, most of the transcripts participate in the cellular process, metabolic process, single-organism process and response to stimulus of BP, the catalytic activity and binding of MF, and the cell, organelle and membrane of CC.

**Fig. 4:**
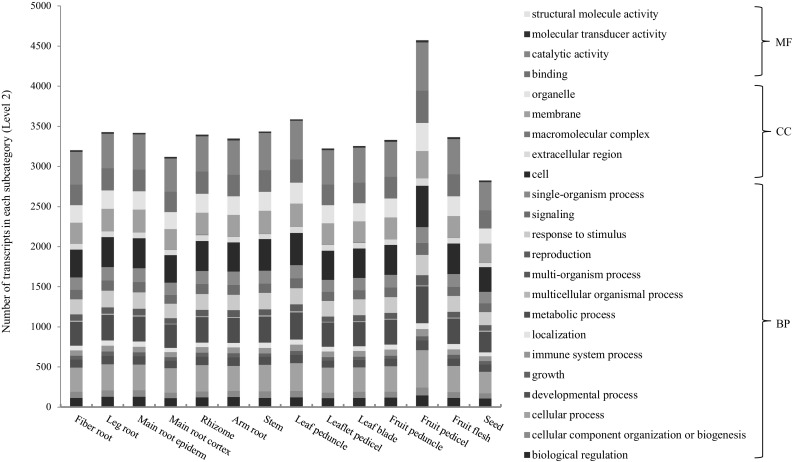
Variation of the functional categories of the *PgRLK* transcripts among 14 tissues of a 4-year-old plant

**Fig. 5:**
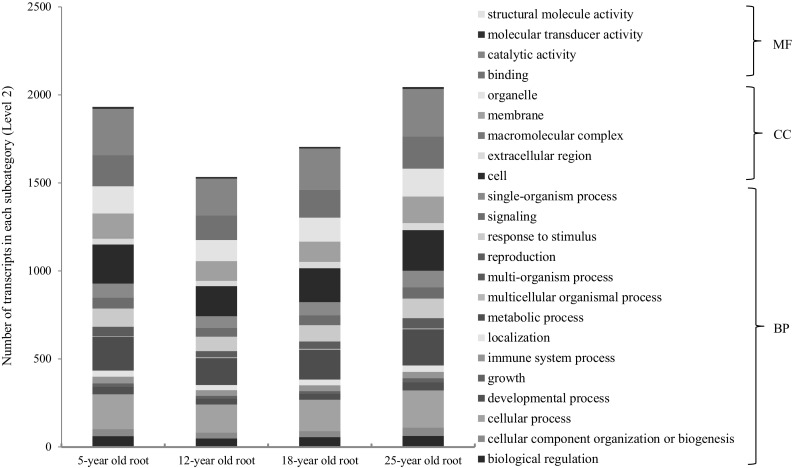
Variation of the functional categories of the *PgRLK* transcripts among the roots of differently aged plants

**Fig. 6:**
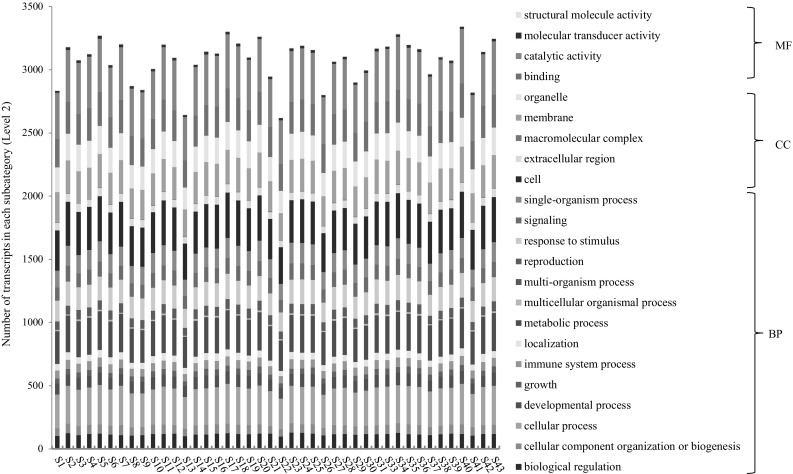
Variation of the functional categories of the *PgRLK* transcripts among the 4-year-old roots of 42 ginseng cultivars from Jilin, China

Furthermore, we categorized the functions of the transcripts of the *PgRLK* genes expressed in 14 tissues (Fig. 4), the roots of four differently aged plants (Fig. 5) and the 4-year-old roots of 42 cultivars (Fig. 6). Although the numbers of the transcripts that were categorized into each subcategory (Level 2) varied dramatically among tissues, among the roots of different-aged plants and among the roots of different cultivars, the transcripts of every tissue, every aged root and every cultivar were all categorized into the 23 subcategories into which the transcripts of all 14 tissues were categorized. These results have further confirmed the functional diversity of the *PgRLK* gene family and also indicated the consistence of functions of the *PgRLK* gene family across tissues, developmental stages and cultivars.

### The expression and expression network of the genes in the *PgRLK* gene family

One may expect that the gene members of a gene family have similar or correlated expressions because they evolved from a common ancestral gene. Therefore, we quantified the expressions of all 964 *PgRLK* transcripts in different tissues, roots from different-aged plants and the 4-year-old roots of different cultivars (Table S6). Figure S2 shows the expressions of the *PgRLK* transcripts categorized into the immune system process. The expression variation of the transcripts among different tissues, different-aged plant roots and the 4-year-old roots of different cultivars ranged from silent (0 TPM) to 400 TPM. These results indicated that as the functions of the *PgRLK* gene family differentiated, the expressions of its gene members have also diverged. This phenomenon was observed not only family wide, but also among the *PgRLK* genes categorized into the same functional subcategory (Fig. S2).

**Fig. 7:**
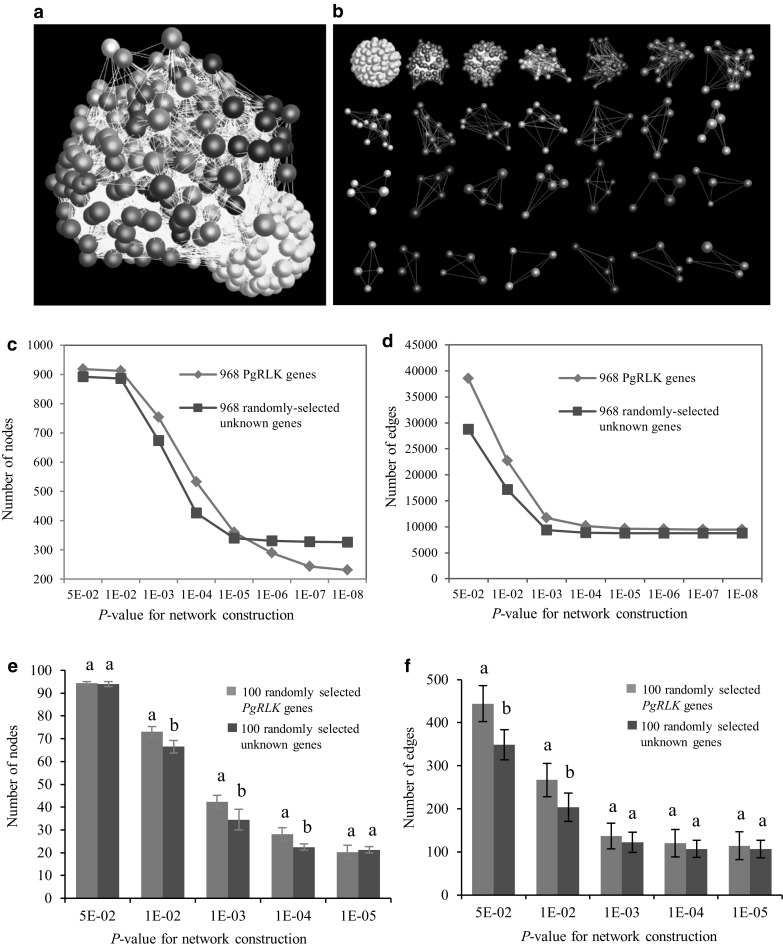
Network analysis of the *PgRLK* transcripts expressed in 14 tissues of a 4-year-old plant. **a** The co-expression network constructed from 920 of the 964 *PgRLK* transcripts. The network was constructed at *P* ≤ 5.0E−02. It consists of 920 gene nodes and 45,328 edges. **b** 28 clusters in the network. **c** Tendency that *PgRLK* genes form a network using the randomly selected ginseng unknown genes as a control: variation in number of nodes. **d** Tendency that *PgRLK* genes form a network using the randomly selected ginseng unknown genes as a control: variation in number of edges. **e** Statistics of variation in number of nodes in the *PgRLK* network. **f** Statistics of variation in number of edges in the *PgRLK* network. Small letters, significant at *P* ≤ 0.05; capital letters, significant at *P* ≤ 0.01; same letters, not significant

**Fig. 8:**
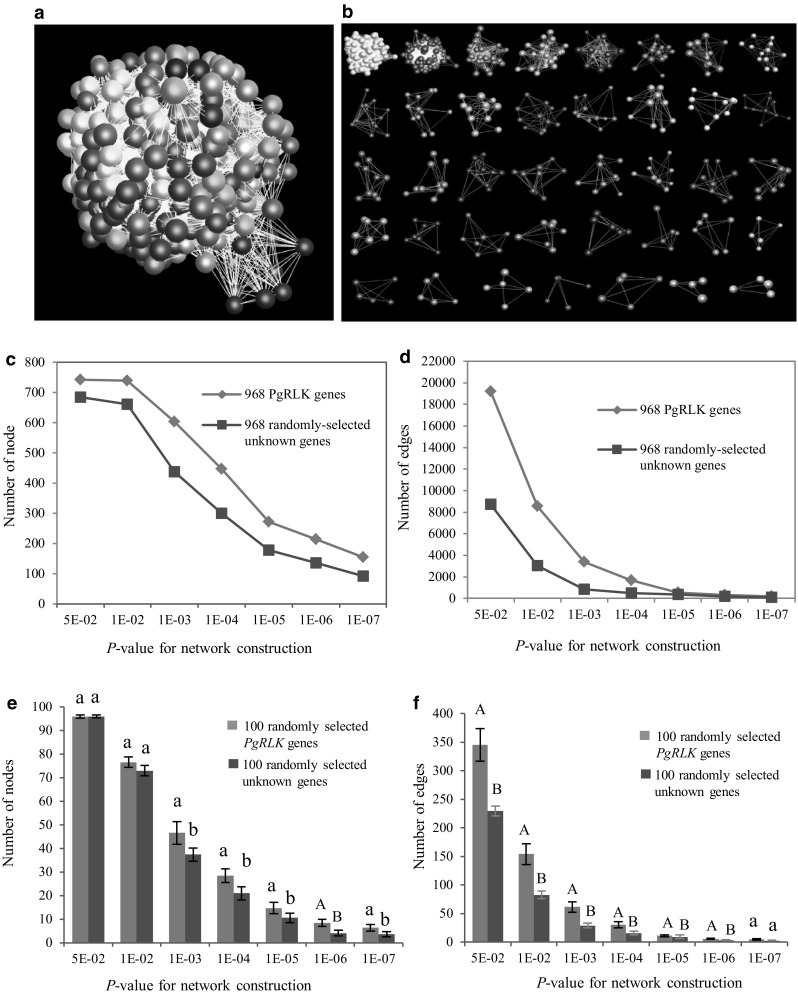
Network analysis of the *PgRLK* transcripts expressed in the 4-year-old roots of 42 cultivars from Jilin, China. **a** The co-expression network constructed from 744 of the 964 *PgRLK* transcripts. The network was constructed at *P* ≤ 5.0E-02. It consists of 744 gene nodes and 26,846 edges. **b** 39 clusters in the network. **c** Tendency that *PgRLK* genes form a network using the randomly selected ginseng unknown genes as a control: variation in number of nodes. **d** Tendency that *PgRLK* genes form a network using the randomly selected ginseng unknown genes as a control: variation in number of edges. **e** Statistics of variation in number of nodes in the *PgRLK* network. **f** Statistics of variation in number of edges in the *PgRLK* network. Small letters, significant at *P* ≤ 0.05; capital letters, significant at *P* ≤ 0.01; same letters, not significant

Therefore, we further examined whether the expression activities of the gene members of the *PgRLK* gene family are somehow related. Unknown ginseng genes randomly selected from the ginseng transcriptome database (Wang et al. 2015) were used as the negative control. In different tissues of a 4-year-old plant, 920 (95.4%) of the 964 *PgRLK* transcripts tended to have correlated expressions and form a co-expression network, relative to the randomly selected unknown genes (Fig. 7a–d). In the same tissue (4-year-old root) of different cultivars, 744 of the 964 *PgRLK* transcripts also had a weak tendency of correlated expression (Fig. 8a–d). This tendency was statistically significant (Figs. 7e,f; 8e, f). These results together indicated that although the functions and expressions of the gene members of the *PgRLK* gene family have dramatically diverged, they do retain a weak tendency of correlated expression and functional cooperation.

### Structural variation, functional differentiation, expression and network of the *PgRLK* gene family

**Fig. 9:**
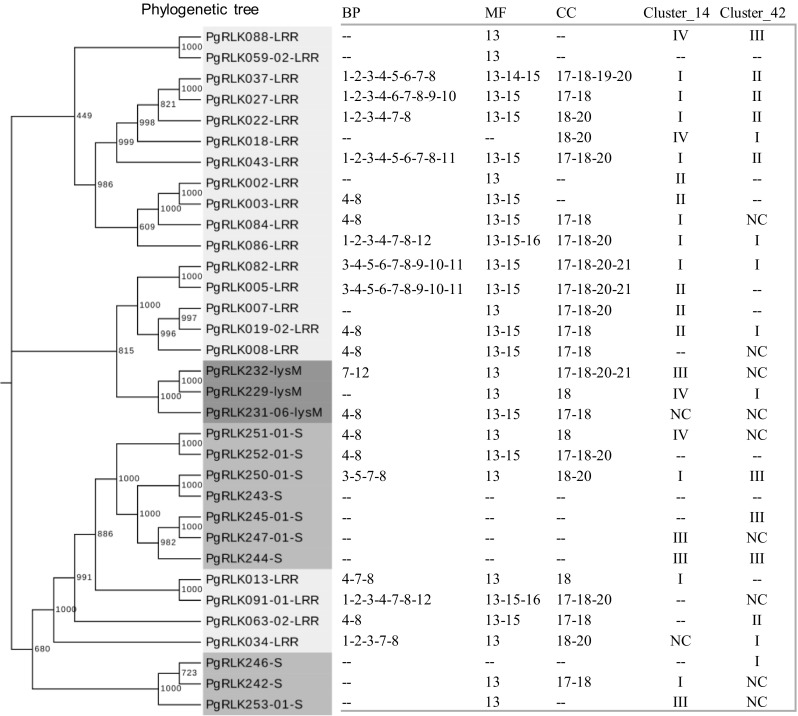
Phylogeny, GO functional categorization and network of the *PgRLK* genes randomly selected from the family tree. The networks of the genes were constructed by their expressions in 14 tissues (Cluster_14) and in the 4-year-old roots of 42 cultivars from Jilin, China (Cluster_42). BP (biological process): 1, biological regulation; 2, signaling; 3, single-organism process; 4, metabolic process; 5, cellular component organization or biogenesis; 6, developmental process; 7, response to stimulus; 8, cellular process; 9, growth; 10, localization; 11, reproduction; 12, immune system process. MF (molecular function): 13, catalytic activity; 14, structural molecule activity; 15, binding; 16, molecular transducer activity. CC (cellular component): 17, organelle; 18, cell; 19, macromolecular complex; 20, membrane; 21, extracellular region. NC, no class; I, II, III, IV, network cluster 1, 2, 3 and 4, respectively. “--”, missing data

**Fig. 10:**
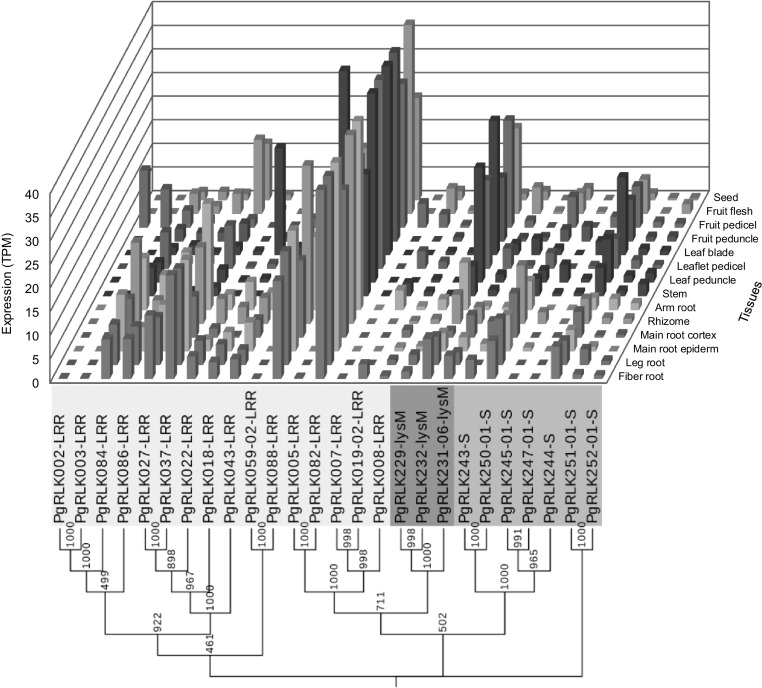
Phylogenetic relationship and expression of the *PgRLK* genes randomly selected from the phylogenetic tree of the *PgRLK* gene family. The figure shows the expression of the *PgRLK* genes clustered into different clusters of the *PgRLK* gene family tree in 14 tissues of a 4-year-old ginseng plant. The number of each branch indicates the bootstrap confidence of the branch with 1000 replications

Finally, we examined the relationships among structural variation, functional differentiation, expression and network of the *PgRLK* genes. The results are shown in Figs. 9 and 10, and Figs. S3–S5. The *PgRLK* genes that were phylogenetically clustered into a single cluster tended to be categorized into the same GO functional subcategory and the same network cluster (Fig. 9). This result indicated that the functional differentiation and network of the *PgRLK* genes were likely to have some correlations with their structural variations (Fig. 9). Nevertheless, when a comparison was made between the gene phylogeny and expression, the expressions of the genes seemed to have negligible relationships with the gene structural variations (Fig. 10, and Fig. S3 and S5). Comparative analysis between gene expressions and network showed that the expression activities of the *PgRLK* genes were related to their network (Fig. S4).

## Discussion

### The *PgRLK* gene family provides a genic resource for advanced ginseng research and genetic improvement

It has been documented that the *RLK* gene family plays important roles in a variety of biological processes, including growth and development, abiotic and biotic stress tolerances, hormone perception and self-incompatibility (Morris and Walker 2003; Shiu et al. 2004; Afzal et al. 2008; Walia et al. 2009; Liebrand et al. 2013; Zhang et al. 2015). However, little is known about this gene family in ginseng. This study has identified the first 563 *RLK* genes that are expressed in Jilin ginseng (*PgRLK* genes). Submission of these *PgRLK* genes to GenBank will fill in the absence of ginseng *RLK* gene resources in it. These gene resources include their nucleotide sequences and also their expression profiles in 14 different tissues, 42 cultivars that represent the ginseng gene resources of the major ginseng origin and diversity center, Jilin, China, and different developmental stages spanning 25 years. Because these *PgRLK* genes were identified from a Jilin ginseng transcriptome database derived from 14 tissues and containing 248,993 transcripts, the *PgRLK* gene resources identified in this study likely contain a strong representation of the *PgRLK* gene family. Moreover, given that the Jilin ginseng transcriptome database was created by shotgun RNA sequencing followed by transcript assembly (Wang et al. 2015), and that the 964 *PgRLK* transcripts have an average length of 1596 bp (Table S1), it is highly likely that the majority of the *PgRLK* genes identified in this study are full length. Therefore, these *PgRLK* genes provide a very useful genic resource for advanced research of the *RLK* gene family in plants and its effective uses for genetic improvement of ginseng and related species.

### The *PgRLK* gene family is a large and diverged gene family

Since the 563 *PgRLK* genes were identified from a transcriptome database and if these expressed genes represent 2/3 of the *PgRLK* genes contained in the ginseng genome (Qi XL, ZMP and Zhang H-B, in preparation), it is estimated that ginseng has approximately 820 *PgRLK* genes. This number of members constitutes a large gene family, but it is not as large as expected, in view of its large genome (3.2 Gb/1C), polyploidy (4*x*) and perennial nature. This number of *RLK* genes is much smaller than those of a number of plant species with even smaller and less complex genomes. For instance, rice (0.4 Gb/1C), poplar (0.5 Gb/1C), soybean (1.1 Gb/1C) and *Arabidopsis* (0.12 Gb/1C) were found to have 600–1700 *RLK* genes (Zhang et al. 2010; Shiu et al. 2004; Lehti-Shiu et al. 2009; Liu et al. 2009).

Phylogenetic analysis in this study shows that the *PgRLK* gene family is found to cluster well with the *RLK* genes cloned from *Arabidopsis*, rice, tomato, potato and carrot. These results suggest that the origin of the *PgRLK* gene family could be traced to before the split between monocotyledons (rice) and dicotyledons (*Arabidopsis*, tomato, potato, carrot and ginseng). The *PgRLK* gene family has diverged into eight types, *PgRLK-*LRR, *PgRLK-*cal, *PgRLK-*pro, *PgRLK-*PERK, *PgRLK-*cys, *PgRLK-*LysM, *PgRLK-*Lec and *PgRLK-*S. Of these eight types of *PgRLK* genes, the LRR type has the largest number of gene members of the family, originated earliest and is most diverged, followed by the S and cys types, and then the LysM and cal types. Although the PERK, Lec and pro types originated most recently, only the Lec type likely originated after *P. ginseng* evolved.

### The functions of the *PgRLK* gene family has substantially differentiated, participating in a variety of biological processes

Consistent with its sequence divergence, the functions of the *PgRLK* gene family have been substantially differentiated. The *PgRLK* gene family participates in at least 23 GO functional subcategories distributed in all three primary GO functional categories, biological process, molecular function and cellular component. Nevertheless, a majority of the genes in the *PgRLK* gene family participate in six GO terms, including cellular process, metabolic process, single-organism process, catalytic activity, binding, and cell and organelle. Functional differentiation is also dominant within each type of the *PgRLK* genes, with the genes of each type participating in at least seven of the 23 GO subcategories of the *PgRLK* gene family.

### The expressions of the *PgRLK* genes vary dramatically among the members of their family, but most of them tend to express correlatively, forming a co-expression network

Although the *PgRLK* genes are from a single gene family and share the conserved domains that specify the *RLK* gene family, their expressions vary dramatically within a tissue, within a genotype or at a developmental stage. The variation of the gene expressions does not seem to be correlated with their phylogenetic relationships and GO terms into which they are categorized. Nevertheless, the *PgRLK* genes that are grouped into the same cluster in the network of the gene family have more similar expression activities than those from different clusters. Also, a gene member that expresses more or less actively in a tissue, in a genotype or at a developmental stage also tends to express more or less actively in other tissues, in other cultivars or at other developmental stages.

While the functionality and expression activities of the *PgRLK* genes have dramatically diversified, most of the genes tend to have correlated expressions, forming a co-expression network even though the correlation is limited. This indicates that the members of the gene family still somehow interact or remain functionally coordinated. However, this interaction is limited, more likely being a remnant interaction, instead of a newly developed interaction. The formation of the numerous clusters within the network of the gene family provides a line of evidence for this inference and further confirms the functional differentiation among the members of the *PgRLK* gene family.

## Electronic supplementary material

Below is the link to the electronic supplementary material.

Supplementary material 1 (PPTX 657 KB)

Supplementary material 2 (PPTX 446 KB)

Supplementary material 3 (PPTX 166 KB)

Supplementary material 4 (PPTX 280 KB)

Supplementary material 5 (PPTX 257 KB)

Supplementary material 6 (XLSX 377 KB)

Supplementary material 7 (XLSX 28 KB)

Supplementary material 8 (XLSX 113 KB)

Supplementary material 9 (XLSX 23 KB)

Supplementary material 10 (XLSX 51 KB)

Supplementary material 11 (XLSX 295 KB)

## Abbreviations

RLK: Receptor-like kinase
*PgRLK*: *Panax ginseng* RLK
NBS: Nucleotide binding site
LRR: Leucine-rich repeat
EGF: Epidermal growth factor
TNFR: Tumor necrosis factor receptor
ORF: Open reading frame
MEME: Multiple EM for motif elicitation
cal: Calcium calmodulin regulated
cys: Cysteine rich
Lec: Lectin-like
LysM: Lysin motif
PERK: Perk1-like
pro: Protein kinase
S: s-domain class
GO: Gene ontology
MF: Molecular function
BP: Biological process
CC: Cellular component
