# Supplementary material for: Evolution, functional differentiation, and co-expression of the RLK gene family revealed in Jilin ginseng, Panax ginseng C.A. Meyer
Source: Mol Genet Genomics. 2018 Feb 21;293(4):845–59. doi: 10.1007/s00438-018-1425-6 (PMC6061065; doi:10.1007/s00438-018-1425-6)
Supplement: Supplementary file 1 — Supplementary material 1 (PPTX 657 KB) [file 438_2018_1425_MOESM1_ESM.pptx]

## Slide 1
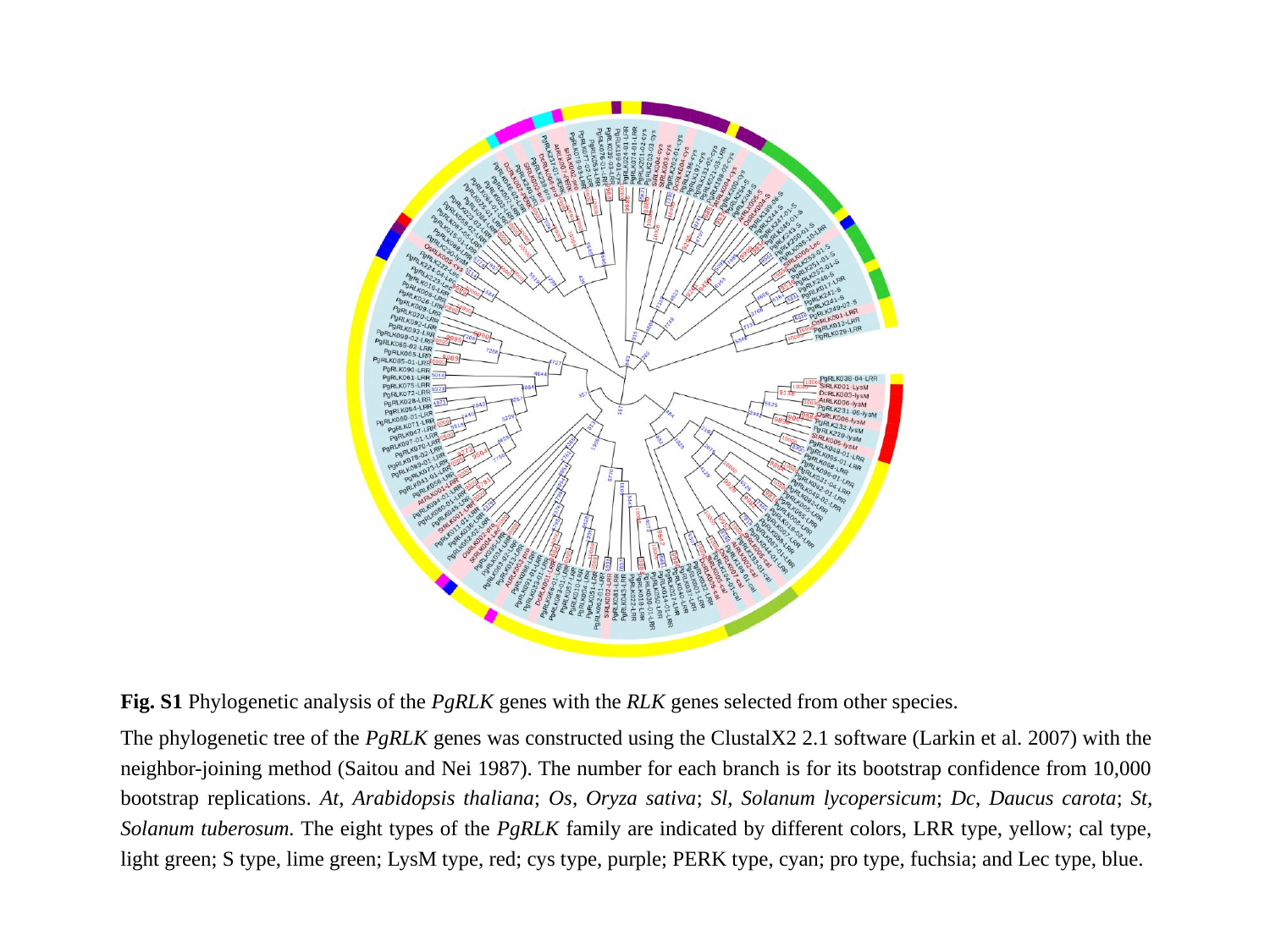

Fig. S1 Phylogenetic analysis of the PgRLK genes with the RLK genes selected from other species.
The phylogenetic tree of the PgRLK genes was constructed using the ClustalX2 2.1 software (Larkin et al. 2007) with the neighbor-joining method (Saitou and Nei 1987). The number for each branch is for its bootstrap confidence from 10,000 bootstrap replications. At, Arabidopsis thaliana; Os, Oryza sativa; Sl, Solanum lycopersicum; Dc, Daucus carota; St, Solanum tuberosum. The eight types of the PgRLK family are indicated by different colors, LRR type, yellow; cal type, light green; S type, lime green; LysM type, red; cys type, purple; PERK type, cyan; pro type, fuchsia; and Lec type, blue.
