# Supplementary material for: Evolution, functional differentiation, and co-expression of the RLK gene family revealed in Jilin ginseng, Panax ginseng C.A. Meyer
Source: Mol Genet Genomics. 2018 Feb 21;293(4):845–59. doi: 10.1007/s00438-018-1425-6 (PMC6061065; doi:10.1007/s00438-018-1425-6)
Supplement: Supplementary file 2 — Supplementary material 2 (PPTX 446 KB) [file 438_2018_1425_MOESM2_ESM.pptx]

## Slide 1
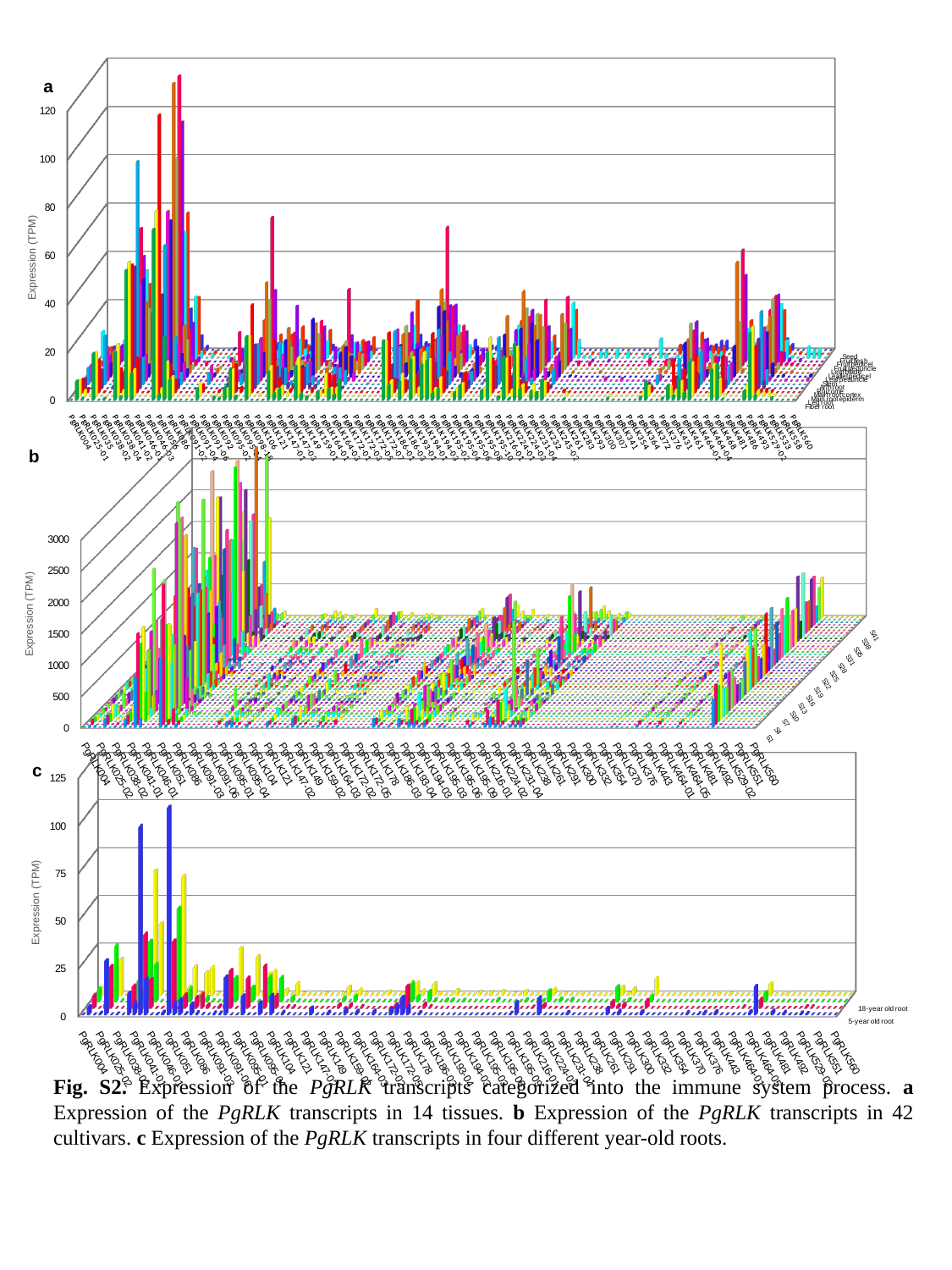

[unsupported chart]
a
Expression (TPM)
[unsupported chart]
b
Expression (TPM)
[unsupported chart]
c
Expression (TPM)
Fig. S2. Expression of the PgRLK transcripts categorized into the immune system process. a Expression of the PgRLK transcripts in 14 tissues. b Expression of the PgRLK transcripts in 42 cultivars. c Expression of the PgRLK transcripts in four different year-old roots.
