# Supplementary material for: Evolution, functional differentiation, and co-expression of the RLK gene family revealed in Jilin ginseng, Panax ginseng C.A. Meyer
Source: Mol Genet Genomics. 2018 Feb 21;293(4):845–59. doi: 10.1007/s00438-018-1425-6 (PMC6061065; doi:10.1007/s00438-018-1425-6)
Supplement: Supplementary file 3 — Supplementary material 3 (PPTX 166 KB) [file 438_2018_1425_MOESM3_ESM.pptx]

## Slide 1
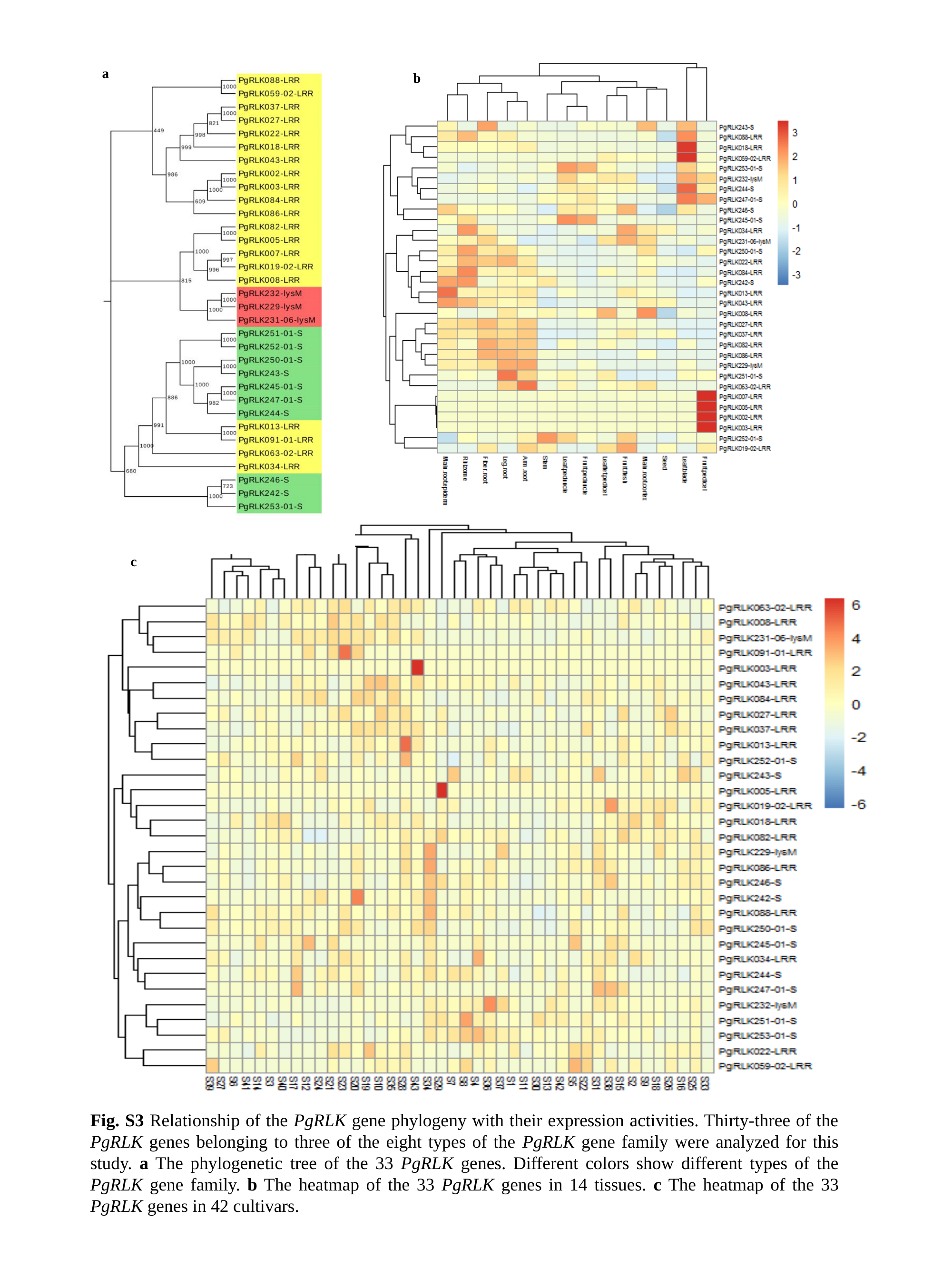

a
b
c
Fig. S3 Relationship of the PgRLK gene phylogeny with their expression activities. Thirty-three of the PgRLK genes belonging to three of the eight types of the PgRLK gene family were analyzed for this study. a The phylogenetic tree of the 33 PgRLK genes. Different colors show different types of the PgRLK gene family. b The heatmap of the 33 PgRLK genes in 14 tissues. c The heatmap of the 33 PgRLK genes in 42 cultivars.
