# Supplementary material for: Evolution, functional differentiation, and co-expression of the RLK gene family revealed in Jilin ginseng, Panax ginseng C.A. Meyer
Source: Mol Genet Genomics. 2018 Feb 21;293(4):845–59. doi: 10.1007/s00438-018-1425-6 (PMC6061065; doi:10.1007/s00438-018-1425-6)
Supplement: Supplementary file 4 — Supplementary material 4 (PPTX 280 KB) [file 438_2018_1425_MOESM4_ESM.pptx]

## Slide 1
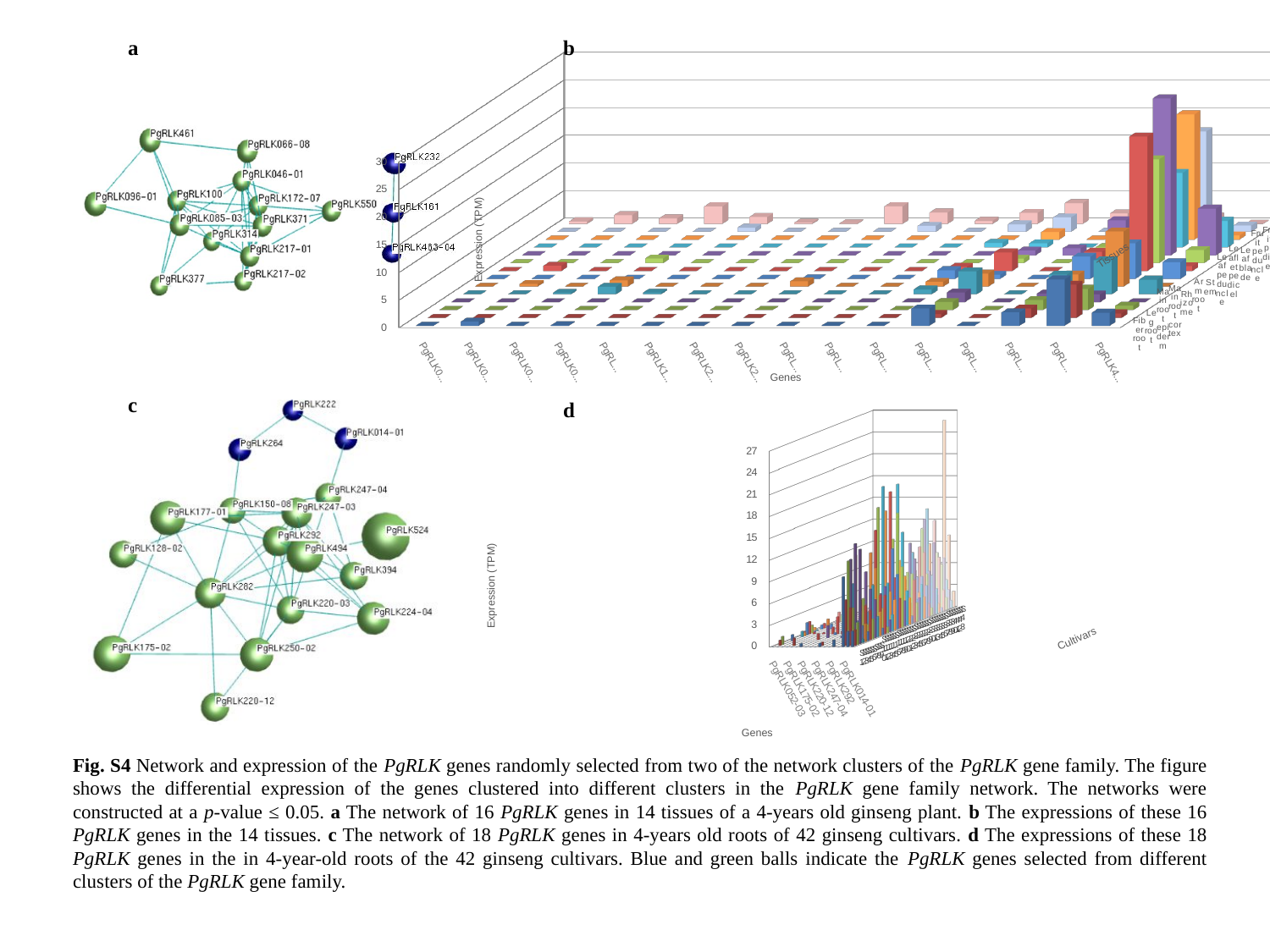

[unsupported chart]
a
b
Expression (TPM)
Tissues
[unsupported chart]
Genes
c
d
Expression (TPM)
Cultivars
Genes
Fig. S4 Network and expression of the PgRLK genes randomly selected from two of the network clusters of the PgRLK gene family. The figure shows the differential expression of the genes clustered into different clusters in the PgRLK gene family network. The networks were constructed at a p-value ≤ 0.05. a The network of 16 PgRLK genes in 14 tissues of a 4-years old ginseng plant. b The expressions of these 16 PgRLK genes in the 14 tissues. c The network of 18 PgRLK genes in 4-years old roots of 42 ginseng cultivars. d The expressions of these 18 PgRLK genes in the in 4-year-old roots of the 42 ginseng cultivars. Blue and green balls indicate the PgRLK genes selected from different clusters of the PgRLK gene family.
