# Supplementary material for: Evolution, functional differentiation, and co-expression of the RLK gene family revealed in Jilin ginseng, Panax ginseng C.A. Meyer
Source: Mol Genet Genomics. 2018 Feb 21;293(4):845–59. doi: 10.1007/s00438-018-1425-6 (PMC6061065; doi:10.1007/s00438-018-1425-6)
Supplement: Supplementary file 5 — Supplementary material 5 (PPTX 257 KB) [file 438_2018_1425_MOESM5_ESM.pptx]

## Slide 1
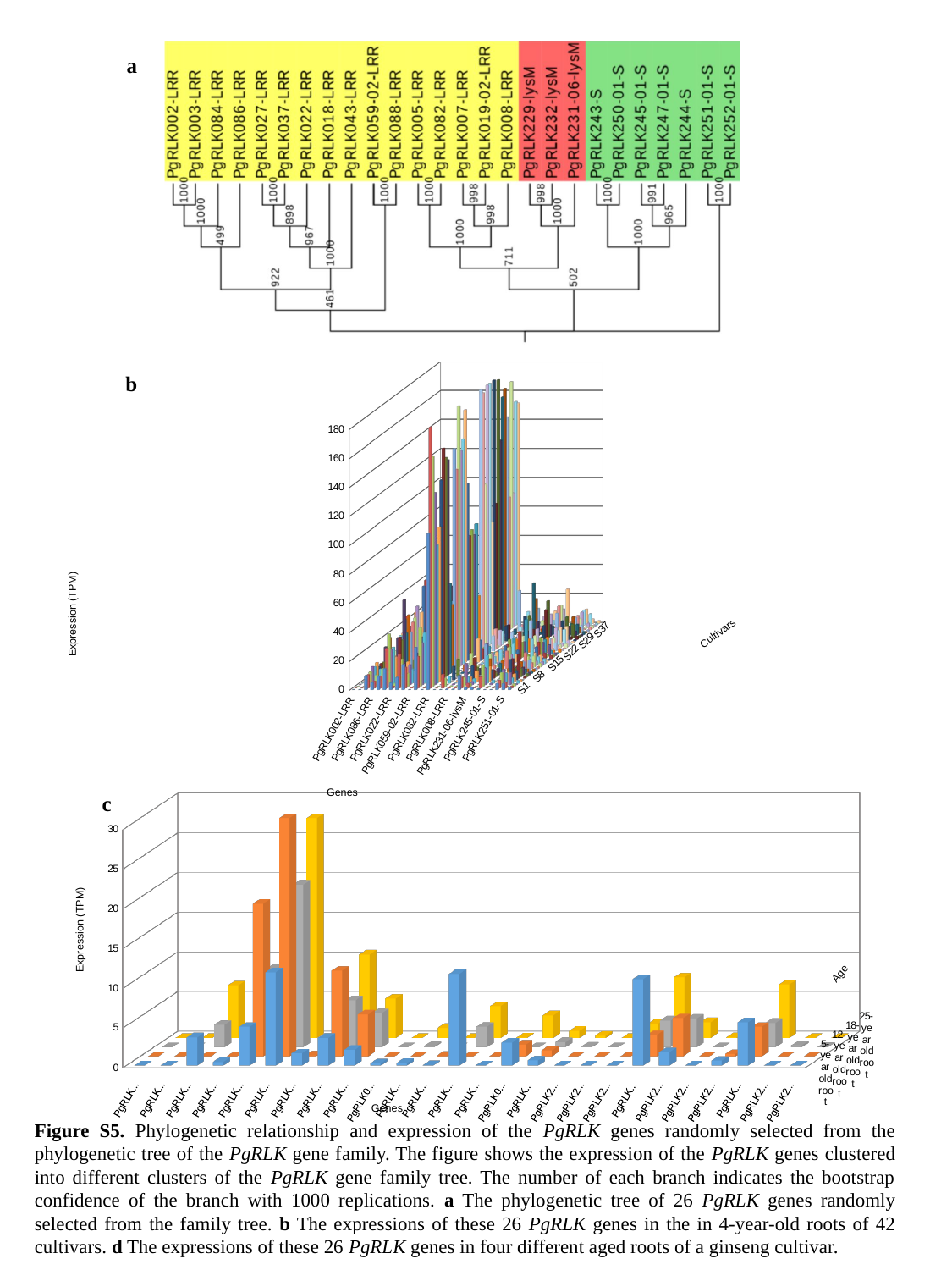

a
[unsupported chart]
b
Expression (TPM)
Cultivars
[unsupported chart]
Genes
c
Expression (TPM)
Age
Genes
Figure S5. Phylogenetic relationship and expression of the PgRLK genes randomly selected from the phylogenetic tree of the PgRLK gene family. The figure shows the expression of the PgRLK genes clustered into different clusters of the PgRLK gene family tree. The number of each branch indicates the bootstrap confidence of the branch with 1000 replications. a The phylogenetic tree of 26 PgRLK genes randomly selected from the family tree. b The expressions of these 26 PgRLK genes in the in 4-year-old roots of 42 cultivars. d The expressions of these 26 PgRLK genes in four different aged roots of a ginseng cultivar.
